# A Little Bug with a Big Bite: Impact of Hemlock Woolly Adelgid Infestations on Forest Ecosystems in the Eastern USA and Potential Control Strategies

**DOI:** 10.3390/ijerph14040438

**Published:** 2017-04-19

**Authors:** Amanda Letheren, Stephanie Hill, Jeanmarie Salie, James Parkman, Jiangang Chen

**Affiliations:** 1 Department of Public Health, The University of Tennessee, Knoxville, TN 37996, USA; alethere@vols.utk.edu (A.L.); shill54@vols.utk.edu (S.H.); jsalie@utk.edu (J.S.); 2 Lindsay Young Beneficial Insects Laboratory, Department of Entomology and Plant Pathology, The University of Tennessee, Knoxville, TN 37996, USA

**Keywords:** hemlock trees, hemlock woolly adelgid, *Adelges tsugae*, pest management and policy

## Abstract

Hemlock woolly adelgid (*Adelges tsugae* Annand, HWA) remains the single greatest threat to the health and sustainability of hemlock in the eastern USA. The loss of hemlock trees leads to further negative impacts on the diversity and stability of ecosystems in the eastern part of North America. It is, therefore, urgent to develop effective control measures to reduce HWA populations and promote overall hemlock health. Currently available individual and integrated approaches should continue to be evaluated in the laboratory and in the field along with the development of other new and innovative methods.

## 1. Background

### 1.1. Hemlock Trees

Hemlock trees are critical ecological components in the eastern USA that provide habitat for aquatic and terrestrial species [1,2]. Hemlocks are coniferous trees with seed cones that are members of the *Tsuga* genus in the *Pinaceae* family. The *Tsuga* genus contains nine species [3]. Five species (*T. chinensis*, *T. diversifolia*, *T. dumosa*, *T. forrestii*, and *T. sieboldii*) are found in Asia and two further species (*T. heterophylla*, and *T. mertensiana*) occur in western North America. The remaining two species *T. canadensis* (Eastern hemlock) and *T. caroliniana* (Carolina hemlock) are native to eastern North America.

Eastern hemlock trees are quite impressive, reaching up to 50 meters (m) in height with trunks of more than 2 m in diameter and a lifespan over 500 years [4]. Carolina hemlocks are approximately 20 m tall, with trunks ranging from 0.5 to 1.0 m in diameter, and populate only a small area of the eastern United States [5]. The density of hemlocks in the forests and national parks not only provides aesthetic appeal, but offers shade to help regulate stream temperatures, habitats, and wood for framing, sheathing, subflooring and pulpwood [1,6].

Hemlocks are slow-growing and long-lived trees. A hemlock stand tends to create an environment that is suitable for its expansion [1]. The soil surface of the stand is kept from drying out under the hemlock canopies. Hemlocks are extremely shade tolerant and eventually outcompete other tree species due to lack of sunlight [1,7]. Given adequate moisture, hemlock trees could easily become dominant or codominant in coniferous and mixed-hardwood forests [1,8,9,10].

### 1.2. Hemlock Woolly Adelgid

The hemlock woolly adelgid (*Adelges tsugae* Annand, HWA), is a small invasive insect of the order Hemiptera that is primarily responsible for the decline of hemlock trees (*Tsuga* spp.) in the eastern USA [2,11,12]. A typical adult HWA individual is less than 1.5 millimeters (mm) long, so attacks can go unnoticed until the HWA fully infests a hemlock tree [5]. HWA uses its mouthparts (known as stylets), to pierce the base of needles on hemlock twigs, penetrating and feeding on xylem ray parenchyma cells to deplete its nutrient reserves. This process either renders the infested hemlock trees more susceptible to other pests or alters their response to environmental stresses [13], although the physiological mechanisms leading to the mortality are still elusive [14,15,16]. Eastern North American hemlocks have evolved chemical defences to protect against chewing insects, but they are highly vulnerable to sap-sucking insect such as HWA infestations [17,18].

## 2. The Introduction of HWA to Eastern North America

HWA was first recorded along the east coast of the USA in Maymont Park, Virginia in 1951 [5]. However, Havill’s group [19] postulated that the introduction of the HWA into Virginia could be traced back to 1911 when a Japanese gardener was hired to create a traditional style Japanese garden with imported exotic ornamental hemlocks for local landowners. To determine the geographic origin of HWA in North America, Havill and associates conducted phylogenetic analysis by comparing the mitochondrial DNA sequences of HWA samples collected from Asia and North America. Eight distinguishable HWA linages that vary in life cycle, historical biogeography, and host specialization are identified. The lineage of western North America demonstrated higher genetic variation than those in eastern North American, Japan, and Mainland China [20,21]. This suggests the colonization of western North America might have occurred prior to the last glacial period by adelgids directly ancestral to those in southern Japan (perhaps carried by birds, rather than through active dispersal of winged adelgid adults, which are not strong enough to travel across long distances like the Bering land bridge in order to colonize western North America). In contrast, only one haplotype was identified in all HWA samples in eastern North America (from Massachusetts to West Virginia) and the haplotype was the same as those collected from hemlock (*T. sieboldii*) of Honshu, Japan, indicating that modern invasion of HWA in eastern North America originates in southern Japan [20,21]. This lack of genetic diversity of HWA in eastern North America, and its ability to reproduce asexually may be beneficial for its colonization because HWA will be more likely to establish without the constraint of finding mates in low population density locations [22].

The extensive spread of HWA since its introduction has caused the significant decline of hemlocks from New England to the southern Appalachians [5]. Since the mid-1990s, Evans and colleagues have monitored HWA infestation levels annually and also the growth of new twigs on 78 permanent hemlock plots in the Delaware Water Gap National Recreation Area. By 2008, approximately 30% of the hemlock trees had died, and it was projected that without effective intervention 80% of hemlocks will die by 2022 [23].

## 3. Life and Reproductive Cycle of HWA

The inoculation of even just a single ovisac could establish and subsequently initiate the next HWA generation (sistens) in approximately 39% of host hemlock trees [22]. The ability of HWA to reproduce at this incredibly rapid rate has put intense stress on the hemlock tree population [24,25]. In eastern North America, HWA is parthenogenetic [24]. Two generations of HWA are produced each year in eastern North America: progrediens (the spring generation that remain on hemlock), and sistens (an overwintering generation) [26]. The progrediens have two forms: a wingless form that remains on the hemlock and a winged form called sexuparae that flies in search of a suitable host spruce tree [27]. Newly hatched nymphs (also known as crawlers) can be dispersed (i.e., via wind or animals), crawl to new growth on the hemlock trees or settle on foliage [5]. In contrast, sexupara, which are rare except where HWA populations are extremely dense, will fly from the originating hemlock trees in search of their primary host, the tigertail spruce (*Picea torano* (Siebold ex K.Koch) Koehne), for sexual reproduction and on which to deposit eggs. This species of spruce is not present in North America, so this portion of the population dies before sexual reproduction occurs [28]. Crawlers will mature by late May and produce woolly ovisacs containing eggs of the sistens generation in the beginning of June. The sistens generation is wingless, hatches in late spring, overwinters, and survives for about nine months. Shortly after the sistens eggs hatch, the first instar nymphs relocate to the base of needles and become dormant (aestivation) in a few days until the fall when the sistens nymphs break dormancy and begin to feed and develop throughout the winter [27,29,30]. The development of sistens takes advantage of the situation in late fall and early spring when few natural enemies are active and hemlocks produce abundant quantities of nutrients [27,29,30,31]. Adult sistens begin to lay approximately 50–300 eggs in late winter or early spring. Adult progrediens typically lay few eggs, but their offspring mature rapidly after hatching.

In Japan, HWA can switch hosts between hemlocks and the spruce during their life cycle. In contrast, the lack of suitable spruce trees in the eastern part of the USA provides a natural defence against the spread of sexuparae. In the northeastern region of the USA, natural causes (the weather, birds and animals) are considered to be the main drivers that keeps the spread of HWA very active and ongoing [27]. Birds and deer not only depend on hemlock trees for shelter and nests but also provide modes of transportation for HWA migration [32].

## 4. Ecological Impacts of HWA in Southern Appalachian Mountains

Hemlock woolly adelgids attack hemlock trees of all ages and sizes, and infested trees seldom recover [33,34]. Environments where leaf litter is found naturally provide functionality for the entire ecosystem to help protect and nourish the soil and enable animals to reproduce and survive. Nutrient inputs, particularly leaf litter nitrogen, can influence soil nitrogen availability [35,36,37,38]. In southern New England, HWA-induced ecological disturbance were associated with the increased N cycling and N turnover rates [39]. In addition, annual nitrification rates were 29 times higher in HWA infested areas than that under healthy forests, implying if nitrate leaching occurs in affected regions, it could lead to freshwater pollution in streambeds and in ravines [39,40].

The impact of HWA infestation on hemlock mortality in particular and on the forest ecological system in general could vary at different geological locations in the eastern USA. Eschtruth et al. [41] reported that HWA infestation in the Delaware Water Gap National Recreation Area (at the northern end of the hemlock range) resulted in a more gradual decline of hemlocks partially due to the severe winter extremes in the northern region. In contrast, the southern region of the USA (including the Shenandoah National Park in Virginia) has experienced a more rapid decline of hemlocks due to HWA infestation [42]. The Great Smoky Mountains National Park (GSMNP), which straddles the border between North Carolina and Tennessee, has more hemlock trees than any other park within the USA and has been greatly affected by infestation of the HWA.

Young hemlock trees between the ages of 75 and 100 years old span across 364,000,000 square meters in the Smoky Mountain Range. Old-growth hemlock trees that are over 100 years old spread across an additional 3,200,000 square meters of land. Since its first report in 2002, HWA in the Great Smoky Mountains has spread throughout the mountain range and infested many hemlock trees of all ages [43]. Nuckolls and colleagues [33] conducted one of the first studies examining the short-term impact of HWA infestations on the hemlock trees in Nantahala Mountain Range of North Carolina. In 2004, the investigators created two sets of plots: the girdled plots in which hemlock tress were girdled by handsaw or chainsaw to sever the cambium, phloem and sapwood, and the infestation plots which comprised of HWA-infested hemlock tress. It was anticipated that a rapid decline of hemlock trees (as indicated by reduced basal area growth and enhanced leaf litter fall) would occur in girdled plots compared to a slower progression of decline in HWA-infested plots. Girdled hemlock trees did decline more rapidly compared to trees in the HWA-infested plots during the first few months [33] but unexpectedly HWA-infested hemlocks then experienced an accelerated decline, and by the third year of infestation, there was no difference in basal area increment between the two sets of plots [33].

Similarly, in the first year of the girdled plot, the leaf litter was 1.5 times greater than that in the HWA infested plots; however, by the second year, the leaf litter became only a third of that in the HWA infested plots. This observation suggests that the hemlock tree decline is progressing more quickly in the southern Appalachians, which will significantly impact carbon and nutrition cycling, and subsequently alter the landscape and function of the forests [33,34]. If hemlocks in the southern Appalachians are replaced by *Rhododendron maximum* L., an evergreen ericaceous shrub that favours soils of high organic content and low nutrient availability, it will restrict the recruitment of other, more productive species into the canopy and dictate future patterns of species regenerations in HWA-infested areas [33,34].

The thinning of the hemlock canopy and subsequent increase of light transmission to understory enables seedling regeneration [39]. Northern red oak (*Quercus rubra* L.) is a common replacement species in the eastern USA for declining hemlock stands. However, oaks may grow more slowly in such situations than in typical oak stands due the reduced mycorrhizal inoculum potential in infested hemlock stands [44]. While the increased eastern hemlock mortality due to HWA infestation would immediately decrease forest water use, the red oak, once established, could consume twice the amount of water [45] and increase summertime water use, reduce aquatic habitat, and decrease stream flow and rates of water input to lakes and reservoirs [45].

Fungi are incredibly important to the forest soil. They decompose lignin in logs and fallen branches and serve as food for creatures that inhabit in the soil [46,47]. It is reported that aboveground infestation by HWA significantly affected rhizosphere processes. Specifically, the reductions in photosynthesis and carbohydrate depletion due to HWA infestation resulted in less fungi colonisation and lower bacterial abundance surrounding fine roots of infested trees [48]. Hemlock woolly adelgid infestations also alter the belowground communities that function to facilitate hemlock tree growth. The significant decline in bacteria load decreases mineral nutrient availability and proves greater difficulty to replant hemlocks in formerly-suitable stands [48].

Shade-tolerance advantages of hemlocks outlast other tree species. Hemlock trees also create understory microclimates for nearly 90 bird species and provide protection for a variety of vertebrate species [5,49]. The black-throated green warbler (*Dendroica virens*) and the blue-headed vireo (*Vireo solitarius*) are hemlock obligates and are only present in forests with hemlocks [5]. Studies further report that HWA infestation could induce hemlock decline resulting in reduced breeding population densities and/or lead to the local extirpation of two hemlock obligates, black-throated green warbler and *Empidonax virescens* Vieillot (Acadia flycatcher) [50].

Salamanders are the most abundant forest-floor vertebrates in the Southern Appalachian Mountains [51,52]. Salamanders are restricted to moist soils and areas for cutaneous respiration [53,54] and for deposition and development of eggs [55]. The hemlock trees and the leaf litter provide such natural microhabitat for salamanders [56,57]. The reduction of litter depth and moisture following timber harvesting results in either the elimination of Appalachian salamander populations or forcing adults to emigrate to adjacent, less suitable forest stands [58], and the population would not fully recover until decades later [51,56,59]. It is possible that smaller canopy gaps and subsequent subtle microclimates alterations due to HWA infestation initially may have minimal impact on the abundance of the salamander population unless significant canopy loss continued. In this case, large canopy gaps would permit increased wind effects and a greater penetration of solar radiation causing the drying of the leaf litter and reduction of moisture content [60], which would be detrimental to the survival of salamanders [61,62].

Even when HWA reach high densities in a hemlock stand, it might take up to several decades for vegetation structure to shift [63,64]. Limited information is available on the dynamics of arthropod communities in response to this change in vegetative structure [65]. Ingwell and colleagues evaluated the impact of eastern hemlock mortality on vegetation and invertebrate diversity and community structure by comparing low-impact (low HWA infestation) and high-impact (high HWA infestation) stands in Connecticut [66]. The high-impact hemlock stands comprised a more diverse suite of understory vegetation including species such as black birch (*Betula lenta* L.), red maple (*Acer rubrum* L.), Canada may flower (*Maianthemum canadense* Desf.), witch-hazel (*Hamamelis virginiana* L.), red oak (*Quercus rubra* L.), and chestnut oak (*Quercus prinus* L.) [66,67]. Correspondingly, arthropod community composition of high-impact hemlock stands was shifted in favour of communities dominated by the orders Orthoptera and Coleoptera (class Insecta) and Collembolans (class Entognatha).

Indicator species analysis demonstrated that ground-running spiders (Family Corinnidae) and sheet-web-building spiders (Family Hahniidae) are significantly associated with low-impact hemlock stands, suggesting that their prey species are less abundant in stands heavily infested with HWA [10,66]. In contrast, no changes of ground-level arthropod diversity were observed between the two stands, indicating species in the studied habitats are either less reliant on specific plant species, or that they exhibit a delayed response to changes in vegetation structure.

## 5. Potential HWA Control and Management Options

The complex life cycle of the HWA, the presence of susceptible hosts, and the lack of natural enemies [68] all contribute to the continuing spread of the HWA in eastern North America. Research studies have been conducted to understand the biology, evolution, ecological impacts of this pest in order to identify an effective management plan [17,69]. Current HWA control measures focus on: cultural control; manipulating hemlock resistance to HWA; chemical treatment and biological control.

### 5.1. Cultural Controls

Cultural controls target the reduction of pest establishment, reproduction, dispersal, and survival. However, few if any, have achieved satisfactory success in containing HWA spread. To reduce invasion of HWA, appropriate barriers should be established to minimize animals from visiting hemlock sites [70]. Public signage systems should be established to provide educational information on HWA in national parks where hemlocks are dominant or co-dominant. Policies to discourage moving plants including global trade in live plants for horticultural use, logs, or firewood from HWA infested areas into non-infested areas should be enforced, specifically between March and June when adelgid eggs and crawlers are abundant [70,71]. For instance, new infestations in isolated areas, such as in the Midwest, usually occur through movement of infested nursery stock. Quarantines have been imposed to reduce or prevent further spread in such areas [72,73].

Increasing tree health is another way of culture control for HWA. Healthy hemlock trees can withstand higher densities of the pest than hemlock trees with low vigour [70]. Removing dead or dying branches from hemlocks can improve overall tree health by promoting new growth, as can applying fertiliser to stimulate the growth and vigour of non-infested trees. Nitrogen fertilisers should not be applied to infested trees however, as nitrogen stimulates the population growth of HWA [74,75]. Increased light has been reported to reduce HWA numbers and the pest’s effects on hemlocks. Studies using artificially infested potted seedlings found that reduced light levels increased HWA densities [76,77]. Moderate light levels appeared to provide the best conditions, however, as they reduced HWA numbers and improved photosynthesis. Canopy density providing moderate light levels also protected seedlings from freeze damage [78]. Researchers suggested that selective thinning of natural hemlock stands may have other benefits such as reduced transpiration resulting in increased soil moisture, and hindrance of HWA movement through the forest canopy [78].

It is generally agreed that water resource availability affects herbivore selectivity and damage [77]. Maintaining soil moisture could ensure optimal growing conditions for hemlock trees to combat HWA infestation [5]. It has been shown that infested hemlock trees survived better in mesic sites versus in xeric site [5,14,79,80]. The impact of water availability (well-watered pots vs. water-stressed pots) on the physiology of hemlocks in the presence and absence of HWA infestation was investigated under a controlled greenhouse environment [77]. Because adelgids are known to cause water stress, it was expected that HWA infestation would exacerbate the decrease in water potential for hemlock trees growing in water-stressed pots. Surprisingly and contradictory to previous reports [14,81], adelgid infestation only decreased water potential of hemlock trees growing in well-watered pots; no similar effect was observed in the water-stressed pots [77]. It is possible that water-stressed trees are already physiologically suitable for the insects. Adelgids, however, need to induce water stress in well-watered trees to improve their suitability [77]. Future long-term investigations are warranted to gain a better understanding of how different abiotic factors could impact the dynamics of HWA population and overall hemlock health [77].

Since HWA are dependent on hemlocks for nutrients, feeding on trees in poor health would affect the ability of the insect to obtain necessary nutrients and subsequently adversely impact their physiological health, reducing the population. This perception is challenged in a naturally infested forest setting without fertiliser or insecticide treatment [82]. In this study, population health of HWA on either lightly (analogous to trees within the Crown Condition Rating Guide Class 1) or moderately (analogous to those within Class 2) affected hemlock trees were evaluated. Compared with moderately impacted trees, HWA collected from lightly impacted hemlocks contained higher levels of carbohydrates, total nitrogen, and amino nitrogen. However, HWA from moderately impacted trees exhibited greater fecundity than those from lightly impacted trees [82]. The results of the study call for caution that simply relying on the HWA physiological indicators (such as levels of carbohydrates, total nitrogen, and amino nitrogen) may not necessarily reflect the overall population health of the HWA population [82].

### 5.2. Manipulating Hemlock Resistance

Manipulating hemlock resistance to HWA is another potential control approach. Searching for naturally resistant trees and developing resistant crosses are considered as promising long-term measures for HWA management [11,83,84,85]. Resistance to HWA has been identified in rare individuals in otherwise adelgid-devastated eastern hemlock stands [11,67,86].

The HWA resistance trait is linked to: lower levels of the lipid hexacosanol and the terpene isobornyl acetate [18]; higher levels of the terpenes α-pinene, α-humulene, β-caryophyllene, and germacrene D in resistant hemlocks; and to trees with thicker epicuticular wax at the point of HWA stylet insertion [87,88]. A small amount of evidence exists that suggest terpenoids in the complex oleoresin may serve as the primary defence of conifers against herbivory due to their ability to inhibit acetylcholinesterase in the neuromuscular junction [87].

Terpenoid content of eastern hemlock foliage from a hemlock stand located at Lake Scranton in Scranton (PA, USA) was monitored to investigate whether variation in terpenoid composition could influence the spatial and temporal feeding preferences of HWA [87]. The special features of this study were that: (1) the sample collection covered two complete generations of HWA life cycle since it spanned a complete annual cycle of eastern hemlock development from bud opening, shoot elongation, shoot maturation, to bud-break at the start of the next growing season; and (2) that samples were collected from “healthy” and “HWA resistant” hemlocks. No significant seasonal variations of myrcene levels were observed in the needles. In contrast, shortly after shoot extension, myrcene and germacrene D were elevated in the immature foliage in spring and became a dominant terpenoid in the leaf cushion over the summer months. In autumn after leaf fall, the levels then decreased to the background levels present in previous year’s growth tissues.

Furthermore, sistens crawlers that settled on new growth estivated whereas progrediens crawlers that settled on new growth immediately began feeding on the immature leaf cushion. Therefore, elevated levels of myrcene and germacrene D in new growth of eastern hemlock leaf cushion tissue might promote mortality in the progrediens generation of HWA [87], in other words render hemlocks “resistant”. On the other hand, seasonal changes in the level of myrcene and germacrene D might have an opposite effect on sistens as the levels of these two compounds are low by the time sistens start feeding in mid-October. Therefore, there would be no adventitious possible toxic effects on sistens [87]. Future investigations are required to understand the link of specific chemical concentrations with resistance status in the context of geographical locations and climatic factors to assist in the development of cultivars with HWA resistance.

Another signature chemical profile found in naturally HWA resistant trees relates to foliar chemistry. Leaves with high levels of nitrogen and potassium tend to have higher HWA densities than other leaves, while high foliar levels of calcium and phosphorous concentrations tend to result in lower HWA densities [89]. This finding reinforces the importance of avoiding applications of nitrogen fertilisers to hemlock trees [74,75]. Both Eastern and Carolina hemlocks are susceptible to HWA yet the Chinese hemlock (*Tsuga chinensis* (Franch.) E. Pritz) is highly resistant to the pest. Therefore, developing resistant crosses between North American and Asian hemlocks may reduce HWA densities in the native range [84,85]. However, the effects of the manipulating hemlock resistance are likely to take many decades to be demonstrated and there are no guarantees that selective breeding would work on a large scale in the field [83,90].

### 5.3. Chemical Controls

Current HWA control is largely focused on using either chemical or biological methods [30,83]. Presently, chemical control is considered the most effective and immediate approach, and is used widely in ornamental and landscape settings. A variety of insecticides are capable of controlling HWA through foliar sprays and systemic treatments [91]. Foliar sprays, such as insecticidal soaps and horticultural oils, have been found effective in controlling HWAs on individual or accessible trees [92]. However, foliar sprays are not a permanent solution; impractical for tall trees and large areas; need to be reapplied every few years; and must cover the entire foliage [5]. Imidacloprid (*N*-{1-[(6-chloro-3-pyridyl)methyl]-4,5-dihydroimidazol-2-yl} nitramide), a chloronicotinyl insecticide, is used for controlling sucking insects, soil insects, termites, and some chewing insects. It is one of the essential insecticides for HWA control in eastern hemlocks in the southern Appalachians [2,93,94,95]. Insecticides containing imidacloprid as the active ingredient can be applied through soil drenching, soil injection, or trunk injection [96]. As a member of neonicotinoid family, imidacloprid blocks nicotinic acetylcholine receptors, leading to the accumulation of acetylcholine, the paralysis of the insects, and ultimate termination of nerve impulses [97,98]. Imidacloprid is delivered to the target pests through ingestion or direct contact [94,98,99,100,101,102,103,104,105]. Imidacloprid is also an effective systemic insecticide. Once incorporated into the soil, imidacloprid has a photolysis half-life between 26.5–229 days [106], which allows its continual availability for uptake by roots [102]. Research has shown after soil application imidacloprid can translocate from the roots to the hemlock foliage and be effective in less than 90 days [95,100,103,107]. Olefin, the metabolite of imidacloprid has higher insecticidal propensity than the parent compound. Concentrations of olefin can persist at relative high level for up to 3 years, and could provide extended HWA suppression post treatment [107].

Benton et al. analysed the levels of imidacloprid and its metabolites in the foliage of hemlocks growing in the GSMNP to understand the longevity of imidacloprid treatments. Four to seven years post basal drench treatment, imidacloprid and olefin were still detectable in more than 65% of branchlets, although the levels were below the LC50 for HWA [93]. While these data demonstrated long-term insecticidal effect of imidacloprid treatment [108], it raises public concern of the potential environmental impact due to its persistence post application [106]. Depending on the application methods used, imidacloprid can be present in detectable concentrations in leaves, vascular fluids, pollen and soils, which could unintentionally target beneficial arthropods in the forest; accelerate declines in populations of honey bees and other pollinators which are a vital part to our food security [109]; decrease the abundance and richness of soil-dwelling organisms; and cause unintentional stress of local microclimates [2,99,108,110,111,112].

In 2013, the European Commission adopted a proposal to restrict the use of three insecticides, including imidacloprid, for two years due to the uncertainty about their chronic risk to ecological system [113]. Imidacloprid could negatively impact insectivorous bird populations. In the Netherlands, population of birds significantly declined in areas with higher surface-water concentrations of imidacloprid [114]. Direct evidence of the disturbance of microenvironment due to imidacloprid application in the eastern region of the national parks is still unclear, however.

Chemical control as a stand-alone treatment is not a feasible option in a forest setting due to geographical constraints, or the height of the trees when bringing in equipment is inconvenient [96]. In addition, trees must be treated individually, which can be very costly and time consuming [5]. The water-solubility of systemic insecticides such as imidacloprid allows for rapid uptake of the chemical throughout the trees. However, considering the close association of hemlock trees with bodies of water, there is a possibility for them to leach out and contaminate aquatic sources and organisms over time [12,115,116].

### 5.4. Biological Controls

The discovery and utilization of effective biological control agents is critical to help restore hemlock forests [5,93]. *A. tsugae* has no known parasitoids and no specific pathogens, so the search for natural enemies is limited to predators [17]. The few predators native to eastern North America reported feeding on HWA do not keep its numbers in check, which has contributed to its rapid spread [12]. Evidence has shown that larvae of *Laricobius rubidus* (LeConte), fed a diet of HWA completed development to the adult stage on *A. tsugae* as well as it did on its primary host *Pineus strobi* Hartig [117]. Therefore, *L. rubidus* has the potential to contribute to the biological control of *A. tsugae* in the eastern United States. *Scymnus suturalis* Thunberg moves among eastern white pine, Scotch pine (*Pinus sylvestris* L.) and eastern hemlock depending on the presence of adelgid prey; however, this predator is not found on eastern hemlock after July [90]. The apparent need by adult *S. suturalis* for a source of adelgid eggs throughout the summer may limit its effectiveness as a predator of *A. tsugae* [90]. Another factor affecting the pest’s biological control is the production of a range of chemicals by certain life stages of HWA. These chemicals (anthraquinone, chrysophanol and its anthrone precursor, chrysarobin) may function as feeding deterrents against predators [118].

An effective biological-control agent should not disturb or disrupt the integrity of other aspects of its native habitat [12]. The ideal candidate predators for HWA should be found in the pest’s native range of Japan, eastern Asia or the pacific northwest of the USA. Up to 50 species of generalist and specialist predators of HWA have been identified in Japan or eastern Asia [119]. Several predators have been tested for biological control potential since 1992 [17,90,119,120,121,122,123,124,125,126,127,128,129,130]. For example, the lady beetle *Sasajiscymnus tsugae*, a native predator of HWA in Japan was initially imported to and studied in New Jersey. Field evaluations demonstrated that *S. tsugae* could establish, locally disperse, and survive heat waves in southern states and severe winters in northern states [17]. Since 1995, over two and a half million *S. tsugae* have been released at more than 400 sites on federal and non-federal lands from South Carolina to Maine [17]. However, to date, this predator has not been proven to provide significant control of HWA in forest settings (James Parkman, personal observation), the recovery rates of hemlock trees were not consistent and the reported number of *S. tsugae* that recovered from the lower crown were relative low [131]. More research is needed to monitor their establishment after release in order to assess the effectiveness and the impact of *S. tsugae* on hemlock trees recovery.

*Laricobius nigrinus* beetles, which are native to the northwestern United States and Canada, and feed exclusively on adelgids, have also been used for the biological control of HWA. After approval for release in 2000, *L. nigrinus* were distributed throughout the invasion range of HWA and isolated populations have established from the southern Appalachians to New England [17,126,129,132]. Unlike *S. tsugae*, there is evidence that *L. nigrinus* can exert substantial mortality on HWA and its preference to feed on HWA over other adelgids, making it a viable biological control candidate [132,133]. *Laricobius osakensis*, a species related to *L. nigrinus*, is a voracious predator of HWA widely spread in Japan. Recently, Arsenault, et al. have shown that *L. osakensis*, reared in the laboratory in North Carolina with little to no prior exposure to field environmental cues, responded preferentially to odours produced by eastern hemlock (regardless if it was infested by HWA or not) and moved promptly onto it [129]. This finding suggests *L. osakensis* relies on volatiles produced by HWA’s host trees to locate potential prey. The phenology of *L. osakensis* is highly synchronous with the life cycle of HWA, making it a good candidate for biological control of HWA in North America [17,134,135].

Recently evidence has emerged indicating imported predator beetles could hybridise in the field with native species of HWA predators. Widespread hybridisation with asymmetrical introgression towards *L. nigrinus* on hemlock has been reported between *L. nigrinus*, the predator introduced from western North America and *L. rubidus* [129,133,136]. Hybridisation could result in the loss of genetic identity [124], and unintentional hybridisation could also lead to the loss of host specificity [129,137,138]; an increase of fitness (heterosis or hybrid vigour) [139]; enhance the fecundity of the hybrid biological control agent [140]; decrease fitness of the native biological control agent due to outbreeding depression or “hybrid breakdown” [139,141,142] or displace the native species, ultimately impairing the efficacy of the biological control program. Thus far, it is believed that hybridization has not, and should not impact predation by either *Laricobius* species [12]. It was reported that *L. osakensis* and *L. nigrinus* will mate but produce only sterile eggs. This is fortunate because *L. osakensis* appears to be a better HWA predator than *L. nigrinus* [143,144].

Ideally, a combination of predators should be used to control HWA populations as opposed to one single species. Flowers and colleagues [121] studied the possibility of using multiple predators for HWA control, specifically using *S. tsugae* and *L. nigrinus*. In the laboratory setting, *S. tsugae* showed increased activity during the day and at higher temperatures, while *L. nigrinus* showed increased activity during the night and in spring-like conditions. In a field study, it was found *S. tsugae* and *L. nigrinus* established two years after release onto eastern hemlocks indicating these two predators can survive and coexist. This is encouraging because they differ temporally in their occurrence and predation of HWA with *L. nigrinus* active from late fall to spring and *S. tsugae* from spring to early summer [145]. These results suggest that multiple-predator species combinations that include the specialist predators with complementary temporal and spatial patterns might be superior than single-species for biological control of HWA [121]. More research is warranted to evaluate the population stability and dynamics of HWA predators once released into the field.

### 5.5. Integrated Control

A long-term, sustainable, and cost-effective approach to control HWA will have to integrate chemical control, biological control, and host-plant resistance methods into one comprehensive management programme [83,146,147]. Such integration is likely be more efficient and provide better control over a wider area as well as prolong hemlock health than would otherwise be possible if only one control method described above were used [12,133,148]. This concept involves using a simple, low rate application of chemical treatment of infested hemlock trees, while simultaneously releasing predators and breeding resistant crosses. The chemical treatment can provide short-term protection as the predator populations establish and grow over time. Chemical protection will become less effective as residual activity declines but, by then, an established predator population may be able to control the chemical-resistant HWA population. As the predator populations control HWA populations, the development of resistant crosses may be capable of saving the Eastern hemlock from extinction [15,81,145,146,148].

## 6. Conclusions

The introduction of the combination of multiple specialist predators is a promising biological intervention strategy. However, how to translate and test a successful laboratory story in the field on a much larger scale at various geographical locations, and how to effectively evaluate predators’ behaviour in the field, including their immigration and emigration propensity in response to prey abundance or intraspecific and interspecific predator cues, is still largely unaccomplished [121]. Furthermore, many “effective” predators tested in the laboratories are descended from a limited, original collection, having been consecutively reared for several generations with continuous exposure to a single prey [149], which may not be reflective of the scenario they will encounter in the field. Predator’s searching patterns can change in response to prey type [121]. Evaluating the combination of biological control with the silviculture treatment of canopy thinning to increase light exposure has merit. Canopy thinning is unlikely to affect performance of the most successful predators, *Laricobius* species; and the combination may result in suppression of HWA populations below damaging levels. Hemlock woolly adelgid remains the single greatest threat to the health and sustainability of hemlock in the eastern USA. Currently along with the development of other new and innovative methods, available individual and integrated approaches should continue to be evaluated not only in the laboratory but specific emphasis should also be focus on their application and effectiveness in the field.
